# Effects of the Replacement of Dietary Fish Meal with Defatted Yellow Mealworm (*Tenebrio molitor*) on Juvenile Large Yellow Croakers (*Larimichthys crocea*) Growth and Gut Health

**DOI:** 10.3390/ani12192659

**Published:** 2022-10-03

**Authors:** Jian Zhang, Yanzou Dong, Kai Song, Ling Wang, Xueshan Li, Beiping Tan, Kangle Lu, Chunxiao Zhang

**Affiliations:** 1 Xiamen Key Laboratory for Feed Quality Testing and Safety Evaluation, Fisheries College, Jimei University, Xiamen 361021, China; 2 College of Fisheries, Guangdong Ocean University, Zhanjiang 524088, China

**Keywords:** *Tenebrio molitor* meal, growth performance, humoral immunity, intestinal health, *Larimichthys crocea*

## Abstract

**Simple Summary:**

Fish meal is the most common protein source in aquatic feeds. The decline of fishery resources and the increased demand have led to a shortage of fish meal resources in recent years. To ensure the sustainable development of the aquaculture industry, it is crucial to find a low-price, high-quality protein source to replace fish meal. In this study, substituting fish meal of large yellow croakers (*Larimichthys crocea*) diets with defatted yellow mealworm (*Tenebrio molitor*) test was carried out. The results showed that the dietary fish meal could be replaced by 15% defatted yellow mealworm in feeds containing 40% fish meal without adversely affecting the growth of large yellow croakers, and to some extent improving the immunity of the organism. Substitution levels of 15% or more are beneficial for digestive enzymes. In addition, the moderate addition of defatted yellow mealworm improves intestinal health by improving the structure and microbial composition of the gut.

**Abstract:**

This study was conducted to investigate the effects of *Tenebrio molitor* meal (TM) replacement for fish meal (FM) on growth performance, humoral immunity, and intestinal health of juvenile large yellow croakers (*Larimichthys crocea*). Four experimental diets were formulated by replacing FM with TM at different levels—0% (TM0), 15% (TM15), 30% (TM30), and 45% (TM45). Triplicate groups of juveniles (initial weight = 11.80 ± 0.02 g) were fed the test diets to apparent satiation two times daily for eight weeks. There was no significant difference in final body weight (FBW) and weight gain rate (WG) among TM0, TM15, and TM30, while TM45 feeding significantly reduced the FBW and WG. Compared with TM0, AKP activity in serum was significantly decreased in TM45, while the TM15 group remarkably increased LZM activity. TM30 showed significantly higher serum C3 levels compared to the TM0 group, while the TM addition groups decreased the C4 levels significantly in the serum. In terms of intestinal histology, the addition of TM increased the height and thickness of the intestinal villus and also increased the thickness of the intestinal muscles significantly. The addition of TM significantly reduced the serum DAO and D-lactate concentrations. The results of 16S rRNA gene sequencing showed that the addition of TM significantly enhanced the relative abundance of Bacilli and *Lactobacillus* and contributed to the decrease in the relative abundance of *Plesiomonas*. In addition, the TM30 and TM45 groups significantly reduced the abundance of Peptostreptococcaceae. Overall, our results indicated that TM could be a viable alternative protein source, 6.7% TM supplantation (replacing 15% FM) in large yellow croaker feed improved humoral immunity and intestinal health with no adverse effects on growth. Furthermore, the replacement of FM with 30% and 45% TM adversely affects growth and humoral immunity.

## 1. Introduction

Aquatic products are rich in high-quality protein, EPA, and DHA, which have beneficial effects on human pathologies [1]. The world population is expected to cross 9.7 billion by the end of 2050 [2]. An unprecedented food crisis will emerge [3]. A recent study has predicted that the aquaculture industry will play a critical role in fulfilling the fast-growing demand for animal protein [4]. In the meantime, the growth of aquatic animals needs a high amount of dietary protein intake [5]. Thus, the supply of protein to aquatic feeds is important to the sustainable development of the aquaculture industry. Currently, fish meal (FM) is the major source of protein in aquatic feeds. With balanced amino acid composition, good palatability, and abundant biologically active compounds, FMs have high nutritional value and digestibility for aquatic animals [6,7]. However, with the rapid development of the aquaculture industry, the increasing FM demand put pressure on marine resources [8,9]. In recent years, the shortage of FM is now a big obstacle to having a sustainable aquaculture industry worldwide. Hence, we pay much more attention to replacing FM with novel protein sources [10].

Some animal and plant protein sources are used to replace FM [11]. Animal protein sources are more widely accepted by fish for their more balanced amino acid profiles [12,13]. Especially, insect protein is a type of animal protein that is environmentally and economically sustainable [14]. At the same time, insects have low greenhouse gas and nitrogen emissions during the rearing process and cause less pollution to the environment [15]. However, the lack of relevant research has hindered the promotion and application of insect protein in aquafeeds. At present, the production scale of domestic insect protein is not large, and the price is slightly higher [16]. Thus, many insect proteins have been applied to the study of aquatic feeds, such as black soldier fly (*Hermetia illucens*) larvae meal [17], housefly maggot (*Musca domestica*) meal [18], and field cricket (*Gryllus bimaculatus*) meal [19].

Currently, Yellow mealworm (*Tenebrio molitor*) meal (TM) is one of the novel insect protein sources which is easily available and in which the culture conditions are simple [20]. The defatted TM has a high crude protein content of up to 63.84%, and its amino acid profile is similar to FM (Table 1). Furthermore, previous studies have indicated that TM contained biologically active substances with anti-tumor, anti-bacterial, antioxidant, and immunomodulatory functions [21,22]. Studies had shown that replacing 50% of FM with defatted TM improved the growth and immunity function of Pacific white shrimp (*Litopenaeus vannamei*) [23]. Diets supplemented with defatted TM improve the survival rate of red seabream (*Pargus major*) [24]. Additionally, defatted TM can replace up to 50% of FM in the diet of largemouth bass (*Micropterus salmoides*) without an effect on growth performance [25]. Thus, defatted TM is likely a high-quality protein source in aquatic feeds.

Large yellow croaker (*Larimichthys crocea*) is the most important marine fish in China, with high nutritional value, delicious taste, and important commercial value [26]. In total, 47% crude protein and 10.5% crude lipid can meet the nutritional requirements of large yellow croakers, which means that a large amount of protein is needed in feed [27]. At present, a high proportion of FM is abundantly used in feed for large yellow croakers, which is highly uneconomical and unsustainable [28]. Therefore, it is imperative to find a suitable replacement for FM and develop a sustainable, efficient, and environmentally friendly feed. Currently, studies related to the replacement of large yellow croaker FM by protein sources such as fermented soybean [29] and soy protein concentrate meal [30] have been reported. However, as a carnivorous fish, animal-source protein has more advantages for large yellow croakers. Furthermore, juvenile large yellow croakers have incompletely differentiated and underdeveloped intestines [31], and the production of large yellow croakers is highly susceptible to high juvenile mortality [32]. Some traditional animal sources of protein used to replace FM result in intestine injury and poor growth performance. Here, the present study selected TM to replace FM in large yellow croaker diets. The effect of TM replacement FM on the growth performance and intestinal health of juvenile large yellow croakers was evaluated.

## 2. Materials and Method

### 2.1. Experimental Diets

Four groups of iso-nitrogenous and iso-lipidic diets were formulated with 0%, 6.7%, 13.3% and 20.0% TM protein content to substitute 0% (control group, FM content: 40%), 15%, 30%, and 45% protein content in fish meal (Table 2). All ingredients were crushed, passed through a 180 µm mesh sieve, then accurately weighed according to the feed formulation and produced as 2 mm puffed pellets using a TES65 twin-screw extruder (Modern Yanggong Machinery Technology Development Co., Ltd., Beijing, China). After drying at 45 °C for 12 h, the mixed fish oil and soybean oil were evenly sprayed onto the surface of the pellets, and all diets were sealed and stored at −20 °C.

### 2.2. Fish and Feeding Trial

This study was conducted in accordance with the Experimental Animal Ethics Committee of the Fisheries College, Jimei University, with the approval number 2011-58. Juvenile large yellow croakers were obtained from a local hatchery (Ningde, China). Before the feeding trial, juvenile fish were temporarily cultured in floating sea cages (4.0 m × 4.0 m × 4.0 m) to acclimate to the experimental condition for 14 days. After the adaptation period, 1800 healthy fish with similar size (11.80 ± 0.02 g) were randomly selected and assigned to 12 floating cages (2.0 m × 2.0 m × 2.0 m) with 150 fish per cage. All experimental fish were significantly satiated by hand feeding twice daily (6:00 a.m. and 6:00 p.m.) using the four experimental diets. Satiation is considered to be reached when most fish stop eating. After each meal, the residual feed in each floating sea cage was collected, respectively, dried, and weighed to exclude the feed intake amount. The feeding trial lasted 56 days. During the feeding trial, the water temperature, pH, dissolved oxygen, and salinity ranged as follows: 20.4–25.8 °C, 8.16–8.65, 6.11–7.97 mg/L, and 27.3–27.9‰.

### 2.3. Sample Collection

Before sampling, all fish were fasted for 24 h and anesthetized with eugenol (1:10,000). The total number and weight of fish in each cage were counted to calculate the survival rate (SR) and weight gain (WG). Twenty fish were randomly selected from each cage. Blood was drawn from the tail vein using a sterile injector, and serum was separated by centrifuging (850× *g*, 10 min at 4 °C) and then stored at −80 °C until analysis. The liver and gut were collected for histological and other analyses.

### 2.4. Analytical Methods

#### 2.4.1. Proximate Composition

The proximate compositions of feed and fish were measured according to the AOAC methods [33]. The moisture was analyzed by drying continuously at 105 °C. The crude protein was determined using the Kjeldahl method after acid digestion. The crude lipid was determined by Soxhlet ether extraction, and the ash content was determined by scorching in a muffle furnace at 550 °C for 8 h. The phosphorus (P) concentration in experimental diets was quantified by inductively coupled plasma atomic emission spectrometry (ICP-OES, Prodigy7, Leeman, Hudson, NH, USA). The samples of feed and protein materials were lyophilized to constant weight, and 30 mg of each sample was taken and placed in 15 mL 6N HCl solution and hydrolyzed at 110 °C for 24 h. Determining amino acid profiles was performed with an automated amino acid analyzer (L-8900, Hitachi, Tokyo, Japan).

#### 2.4.2. Digestibility Enzyme

Intestine tissues were homogenized in the pre-cooled normal saline and the homogenate was collected by centrifuging at 3000× *g* for 10 min. Intestinal lipase and trypsin activities were measured by competition method using commercial kits (Nanjing Jiancheng Biological Company, Nanjing, China) according to the manufacturer’s instructions. Quantification of proteins was performed with a total protein quantitative kit (Beijing Solarbio Science and Technology Company, Beijing, China) according to our previous study [34].

#### 2.4.3. Serum Biochemical Parameters

Serum complement 4 (C4) levels, complement 3 (C3) levels, and D-lactate concentration were determined by the competition method according to previous reports [35] using ELISA kits for the fish (Nanjing Jiancheng Biological Company, Nanjing, China). The determination of serum diamine oxidase (DAO), alkaline phosphatase (AKP), and lysozyme (LZM) activities according to the colorimetric method was performed with commercial kits (Nanjing Jiancheng Bioengineering Institute, Nanjing, China). One LZM activity unit was to be defined as the amount of enzyme required to decrease absorptivity at 37 °C at a rate of 0.001 min/mL, and one AKP activity unit was defined as the production of 1 mg of phenol by the action of serum with the substrate for 15 min at 37 °C, and one DAO activity unit was defined as the formation of 1 mmol of ammonia per minute per milliliter of serum at 37 °C.

#### 2.4.4. Intestinal Histology

Gut samples were stained with hematoxylin and eosin (H and E) according to the methods described in our previous studies [36]. Observation of the sections was conducted under a light microscope (Leica DM5500B, Solms, Germany), and morphometric analysis was performed with Image J software (NIH, Bethesda, MD, USA).

#### 2.4.5. Gut Microbiota Collection and Analyses

The extraction of bacterial DNA from a large yellow croaker gut was performed using the HiPure Soil DNA Kit (Magen, Beijing, China), then Nanodrop 2000 (Thermo Scientific, Waltham, MA, USA) was used to detect the DNA yield. Amplicons of the V3-V4 region of the 16SrRNA gene were extracted from a 2% agarose gel and purified using the AxyPrep DNA Gel Extraction Kit (Axygen Biosciences, San Francisco, CA, USA). Quantification was then performed using the ABI StepOnePlus Real-Time PCR System (Life Technologies, New York, NY, USA). The purified amplicons were sent to Illumina Miseq PE 250 for sequence analysis, performed by Gene Denovo Biotechnology Co., Ltd., Guangzhou, China.

All chimeric tags were removed with QIIME software to obtain valid tags for further analysis. The valid tags were clustered into operational taxonomic units (OTUs) of ≥97% similarity using UPARSE software. The highest abundant tags sequence was selected as the representative sequence within each cluster. The biological classification of representative sequences was performed using the RDP classifier based on the SILVA database with a confidence threshold of 80%. An NMDS plot with an unweighted uniFrace distance matrix was used to represent the variability of the intestinal bacterial community structure. LEFSE analysis was performed to compare gut microbial community composition among all groups.

**Table 2:** Formulation and proximate composition of experimental diets (% dry matter).

Ingredients (%)	Experimental Diets			
	TM0	TM15	TM30	TM45
Fish meal	40.00	34.00	28.00	22.00
Chicken meal ^a^	13.00	13.00	13.00	13.00
Soybean meal	10.00	10.00	10.00	10.00
TM ^b^	0.00	6.70	13.30	20.00
High gluten flour	23.00	21.39	19.87	18.25
Soybean oil	1.50	1.50	1.50	1.50
Fish oil	2.50	2.90	3.30	3.70
Lecithin	1.50	1.50	1.50	1.50
Stickwater ^c^	6.00	6.00	6.00	6.00
Vitamin C ^d^	0.10	0.10	0.10	0.10
Vitamin premix ^e^	0.40	0.40	0.40	0.40
Mineral premix ^f^	0.50	0.50	0.50	0.50
Ca(H_2_PO_4_)_2_	1.00	1.50	2.00	2.50
Choline chloride	0.50	0.50	0.50	0.50
Methionine	0.00	0.01	0.03	0.05
Proximate analysis				
Dry matter	98.02	98.49	98.59	98.85
Crude protein	47.98	48.30	48.00	48.10
Crude lipid	8.25	8.63	8.76	8.79
Ash	11.12	10.70	10.85	10.50
Methionine	2.60	2.64	2.67	2.71
Lysine	0.93	0.93	0.93	0.94
Total Phosphorus	1.38	1.38	1.38	1.37

^a^ Chicken meal was supplied by Qingdao Bio-ways Ingredients Bio-technology Co., Ltd., Qingdao, China. ^b^ TM (*Tenebrio molitor* meal) was supplied by Guangzhou Z and C Biology Co., Ltd., Guangzhou, China. ^c^ Stickwater was supplied by Fujian Hongwei Bio-technology Co., Ltd., Zhangzhou, China. ^d^ The form of Vitamin C is L-Ascorbate-2-phosphate. ^e,f^ Vitamin premix and Mineral premix were prepared in our previous work [37].

### 2.5. Calculations and Statistical Analysis

Survival rate (SR, %) = final body number/initial body number × 100

Weight gain rate (WG, %) = (final wet body weight − initial wet body weight)/initial wet body weight × 100

Feed efficiency (FE) = (final wet body weight − initial wet body weight)/dry feed intake

Feed intake (FI, %/day) = total dry feed intake × 2/[(final wet body weight + initial wet body weight) × number of feeding days] × 100

Protein efficiency ratio (PER) = wet body weight gain/dry protein intake × 100

Condition factor (CF, g/cm^3^) = final wet body weight/(final body length)^3^

Hepatosomatic index (HSI, %) = liver wet weight/final wet body weight × 100

Viscerosomatic index (VSI, %) = visceral wet weight/final wet body weight × 100

Protein deposition rate (PDR, %) = (final wet body weight × crude protein in final body − initial wet body weight × crude protein in initial body)/crude protein in diet × 100

Lipid deposition rate (LDR, %) = (final wet body weight × crude lipid in final body − initial wet body weight × crude lipid in initial body)/crude lipid in diet × 100

All data were analyzed by one-way ANOVA using SPSS 22.0, and multiple comparisons were performed using Tukey’s test. *p* < 0.05 is set to indicate a significant level of difference. The data are presented as means ± S.E.M. (standard error of the mean).

## 3. Results

### 3.1. Growth and Feed Utilization

The growth performance, feed utilization, and morphological parameters of the large yellow croaker were shown in Table 3. Compared with the TM0 group, FBW, WG, and FE were significantly lower in the TM45 group. However, there were no significant differences in FBW and WG between the TM0, TM15, and TM30 groups. There was no significant difference in the FE between the TM30 and TM45 groups, and it was significantly lower than the TM0 and TM15 groups. The PER of the TM45 group was significantly lower than that of the TM0 and TM15 groups. There were no significant differences in FI, CF, HSI, and VSI among the groups. In terms of the nutrient deposition rate, the PDR of the TM45 group was significantly lower than the other groups, while there was no significant difference in the PDR between the TM0, TM15, and TM30 groups. Additionally, the LDR showed a decreasing trend from the TM0 group to the TM45 group, and the LDR in the TM30 and TM45 groups was significantly lower than in the TM0 group.

### 3.2. Body Composition

The contents of moisture, crude protein, crude lipid, and ash of the whole-body and liver lipid content were shown in Table 4. The moisture of the whole body was significantly higher in the TM45 group compared to the TM0 group. Additionally, the liver crude lipid in the TM15 group was significantly lower than in the other groups.

### 3.3. Digestion Enzyme

**Figure 1:**
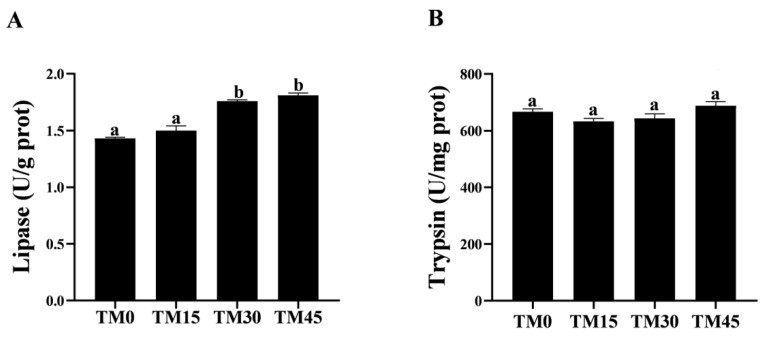
Digestion enzyme activity in the gut of large yellow croakers fed diets with different levels of TM (*Tenebrio molitor* meal): (**A**) lipase and (**B**) trypsin. Bars with different letters (a, b) indicate a significant difference between experimental diets by Tukey’s test (*p* < 0.05).

The gut lipase activity increased with the increasing levels of TM substitution. The lipase activity in the TM30 and TM45 groups was significantly higher than in the TM0 and TM15 groups (Figure 1A), while there was no significant difference in the lipase activity between the TM30 and TM45 groups (Figure 1A). In addition, TM did not significantly affect the gut trypsin activity (Figure 1B).

### 3.4. Non-Specific Immunity

As shown in Table 5, AKP activity in the TM45 group was significantly lower than in other groups. Serum LZM activity in the TM15 group was significantly lower than the TM0 group, but not significantly different from TM30 and TM45. Serum C3 content was significantly higher in the TM30 group than in the TM0 and TM15 groups. Serum C4 content was decreased with increasing levels of substitution, with the lowest level in the TM45 group and significantly lower than the other treatments.

### 3.5. Gut Morphology

**Figure 2:**
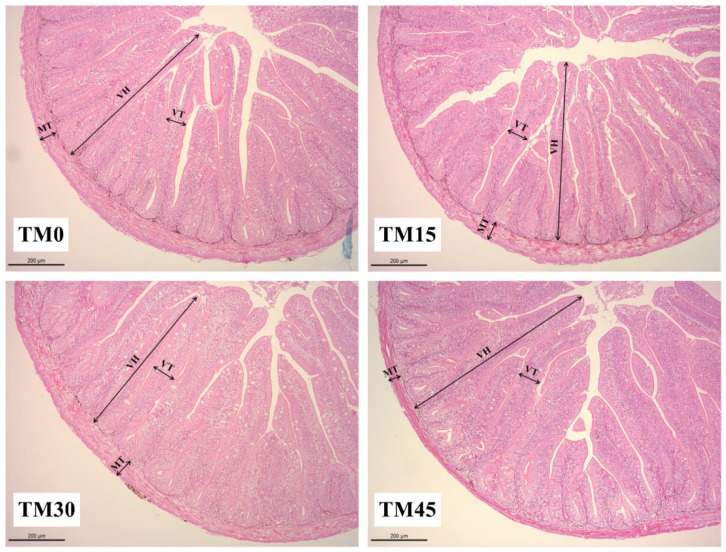
Observation of the gut morphology of large yellow croakers fed diets with different levels of TM (*Tenebrio molitor* meal) by H and E staining (200×). Abbreviations: VH, Villus height; VT, Villus thickness; MT, Muscularis thickness.

**Figure 3:**
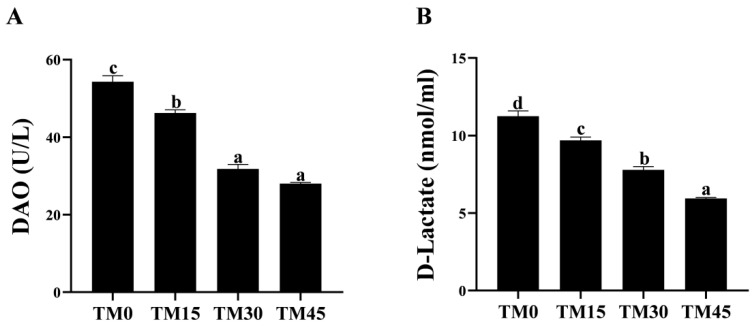
Gut epithelial permeability of large yellow croakers fed diets with different levels of TM (*Tenebrio molitor* meal): (**A**) diamine oxidase (DAO), and (**B**) D-lactate. Bars with different letters (a, b, c, d) indicate a significant difference between experimental diets, by Tukey’s test (*p* < 0.05).

The gut morphology was shown in Figure 2. As shown in Table 6, the gut villus height was the longest and significantly higher in the TM45 group than in the other treatments. The intestinal villus thickness was increased with the increasing levels of diet TM substitution and was significantly higher in the TM30 and TM45 groups than in the TM0 and TM15 groups. There was no significant difference in the muscular thickness of large yellow croakers intestines between TM15 and TM30, but both were significantly higher than the TM0 and TM45 groups. Moreover, the addition of TM significantly reduced the activity of serum DAO, an indicator of the intestinal epithelial physical barrier, but there was no significant difference between the TM30 and TM45 groups (Figure 3A). The serum D-lactate levels showed a downward trend with the TM substitution levels, and the serum D-lactate levels in the TM15, TM30, and TM45 groups were significantly lower than in the TM0 group (Figure 3B).

**Table 6:** Gut morphological indices of large yellow croakers fed diets with different levels of TM.

Index (μm)	Experimental Diets			
	TM0	TM15	TM30	TM45
VH	588.59 ± 10.57 ^a^	620.82 ± 5.70 ^b^	606.52 ± 4.32 ^ab^	664.46 ± 4.95 ^c^
VT	72.56 ± 0.95 ^a^	93.85 ± 2.25 ^b^	104.69 ± 2.02 ^c^	110.06 ± 1.99 ^c^
MT	69.08 ± 0.73 ^a^	76.65 ± 0.87 ^b^	73.64 ± 0.84 ^b^	69.00 ± 0.89^a^

In the same row, values with different superscripts (a, b, c) indicate significant difference (*p* < 0.05). Abbreviations: VH, Villus height; VT, Villus thickness; MT, Muscularis thickness.

### 3.6. Gut Microbial Communities

**Figure 4:**
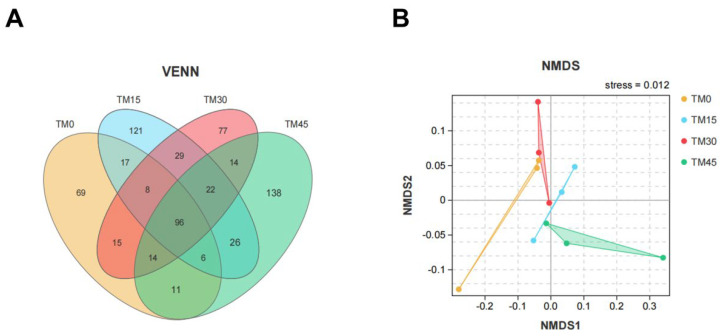
(**A**) Venn diagram, (**B**) non-metric multidimensional scale analysis (NMDS) based on unweighted uniFrac distance matrix of gut microbiota.

**Figure 5:**
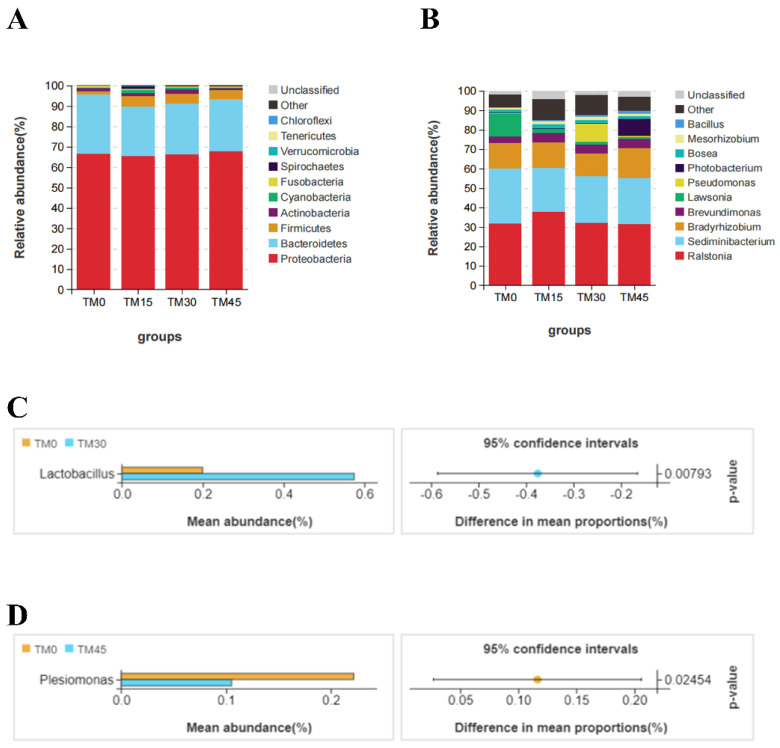
Barplot (**A**) and barplot (**B**) were used to represent the relative abundance of the major intestinal bacterial phyla and genera, respectively. Differences in (**C**) *Lactobacillus* and (**D**) *Plesiomonas* were calculated by Welch’s *t*-test (*p* < 0.05).

**Figure 6:**
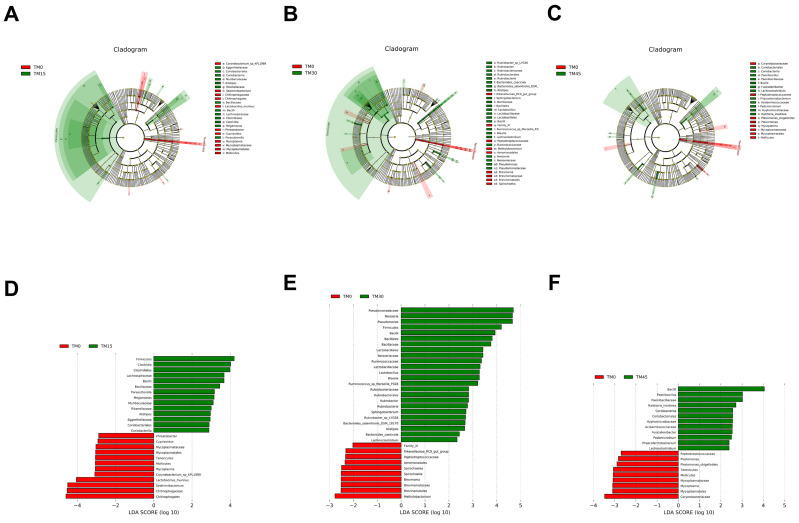
LEFSE (Linear discriminant analysis Effect Size) analysis identified bacterial species that differed significantly in abundance among groups. (**A−C**) Differences in bacterial species abundance were represented by the color (Red indicates a taxon with significantly higher relative abundance in the TM0 group, and green indicates a taxon unit with significantly higher abundance in the TM addition groups). (**D−F**) Linear regression analysis (LDA) was used to estimate the magnitude of the effect of the abundance of each bacterial species on the differential effect.

The Venn diagram of microbial communities showed that the number of core OTUs in the four groups of microbial communities was 96, and the total number of OTUs for the TM0, TM15, TM30, and TM45 groups were 236, 325, 275, and 327, respectively (Figure 4A). The NMDS plot showed no overlapping parts between any of the four groups (Figure 4B). Proteobacteria, Bacteroidetes, and Firmicutes are the major intestinal bacterial phyla of large yellow croakers (Figure 5A). Analyzed from the genus level, *Ralstonia*, *Sediminibacterium,* and *Bradyrhizobium* are the major intestinal bacterial genera of large yellow croakers (Figure 5B). TM30 diets can significantly increase the abundance of Lactobacillus (Figure 5C). The abundance of Plesiomonas was significantly reduced when the substitution level reached 45% (Figure 5D). The results of LEFSE analysis showed that there were significant differences in the bacterial categories of intestinal microbial communities between the control and TM-added groups (Figure 6). TM significantly improved the relative abundance of the class Bacilli in the intestinal tract of the large yellow croakers (Figure 6A–F). In addition, the TM substitution of 15% FM in the diet significantly improved the relative abundance of *Parasutterella*, *Megamonas*, and *Alistipes* (Figure 6A,D). Additionally, when the substitution level reached 30%, the abundance of *Lactobacillus*, *Neisseria*, *Pseudomonas*, *Blautia*, *Sphingobacterium*, *Alistipes*, *Lachnoclostridium*, and *Rubrobacter* in the fish intestine increased (Figure 6B,E). When the substitution level reached 45%, the abundance of *Paenibacillus*, *Pedomicrobium*, *Phascolarctobacterium*, *Lachnoclostridium*, and *Fusicatenibacter* increased (Figure 6C,F).

## 4. Discussion

In this study, the replacement of 30% FM with TM resulted in no obvious difference in the growth performance of fish. The result indicated that TM could be used as a partial substitution for FM. However, TM significantly affected WG when the substitution level reached 45%. Similarly, a study on African catfish (*Clarias gariepinus*) showed that the growth performance of the fish was significantly reduced when the TM substitution level exceeded 40% [38], and a study on European perch (*Perca fluviatilis*) found a decreased growth performance when TM substituted 50% FM [39]. Furthermore, in the present study, FE, PER, LDR, and PDR were reduced in the TM30 and TM45 groups, indicating that high levels of substitution affected feed utilization. In fact, TM contains about 4.92% of chitin [40]. Some studies have confirmed that a diet containing more than 1% chitin will reduce the growth performance of fish [41], which may be one of the reasons for the reduced growth performance of large yellow croakers in the high TM group in this experiment. The above results indicate that replacing 15% FM with TM in the ration is a reasonable substitution level with no adverse effects on the growth performance of juvenile large yellow croakers.

The addition of TM to diets did not affect the crude protein and crude lipid content of the whole body but increased the moisture and ash content, which is consistent with the results of the largemouth bass (*Micropterus salmoides*) study [42]. In addition, the crude lipid content of the liver was reduced in the TM15 group. However, studies in largemouth bass (*Micropterus salmoides*) [41] showed a TM substitution of up to 50% FM in the diet did not affect liver crude lipid content. This needs further study in the future.

Fish humoral immunity is an important component of fish immunity, while serum immune parameters can reflect the immune function of fish [43]. AKP had been proved to have immunomodulatory effects by inhibiting neutrophil production and inflammatory responses [44]. The decreased AKP activity in serum leads to the decreased immunity of fish [45]. LZM is produced by neutrophils and macrophages and secreted into the blood and mucus to exert bacteriolytic effects with antiviral, antibacterial, and anti-inflammatory properties [46]. Furthermore, complement is an important component of fish resistance to microbial infection and is involved in target cell lysis and the regulatory responses of the organism after initiation, while complement 3 and complement 4 are the main components of the complement system [47]. Increasing the serum levels of C3 and C4 improves immune regulation, neutralizes toxins, and blocks inflammatory responses [48]. The results of the present study showed that replacing 45% of FM with TM in diets reduced AKP activity. Replacing 15% of FM with TM increased LZM activity, and replacing 30% of FM increased C3 contents while replacing 30% or more FM decreased C4 contents. These results indicate that the high-level addition of TM leads to a decrease in the immunity function of the large yellow croaker.

It is widely accepted that intestinal histology is a required indicator to assess tissue alterations caused by different diets [49]. Histological analysis through HE straining is a direct way to assess the health of the intestine. It is generally believed that the longer the height and thickness of the intestinal villi, the greater the digestion and absorption capacity of the intestine for nutrients by increasing the contact area with nutrients [50], and muscle stenosis may cause intestinal inflammation to harm intestinal health [51]. In the present study, TM diets increase the thickness of the intestinal villi in large yellow croakers; 15% and 45% substitution levels increase the height of the intestinal villi, and 15% and 30% substitution levels increase the thickness of the muscularis. These results suggest that TM may have a beneficial effect on intestinal health. Similar results were also observed in a previous study that used largemouth bass (*Micropterus salmoides*) [42]. Serum concentrations of D-lactate and DAO often increase when there is damage to intestinal epithelial structures [52]. In the present study, we evaluated intestinal epithelial permeability by measuring serum D-lactate and DAO levels/activity. As a result, TM reduced the serum concentrations of D-lactate and DAO, suggesting that TM has a repairing effect on the intestinal epithelial mucosa of large yellow croakers.

To a certain extent, digestive enzyme activity reflects the degree of the digestion and absorption of feed materials and nutrients by the organism [53]. In the present study, TM did not affect the intestinal trypsin activity of large yellow croakers, but 15% and 45% substitution levels increase the intestinal lipase activity. Research has been conducted to show that fatty acids in insect proteins are incompatible with the digestion of *Pangasianodon hypophthalmus* [54] and that fatty acids act as signaling molecules to regulate lipid metabolic pathways such as lipase [55], which may account for the elevated intestinal lipase activity in the TM high-substitution-level group. The above results indicate that TM has a promotive effect on the intestinal lipase enzymes of large yellow croakers.

Gut flora can influence the physiological, nutritional, immune, and metabolic functions of the host and plays a crucial role in the health status of marine fish [56,57]. In the present study, the three major phyla in the gut of large yellow croakers were *Proteobacteria*, *Bacteroidetes*, and *Firmicutes*. *Ralstonia*, *Sediminibacterium*, and *Bradyrhizobium* are the three major genera of bacteria. The relative abundance of *Pseudomonas* was higher in TM30 and TM45 compared to TM0, and the former was shown to be a fish pathogen [58]. This suggests that high levels of TM can adversely affect the composition of the intestinal flora of large yellow croakers. A previous study found that probiotic bacteria in *Lactobacillus* are beneficial in aquaculture to protect fish from pathogens [59,60,61]. In this study, TM30 increased the abundance of the genus *Lactobacillus*. In contrast, bacteria in *Plesiomonas* are a pathogen in fish, and human accidental ingestion of fish containing this bacterium is highly susceptible to gastrointestinal-related diseases [62,63]. Currently, the abundance of *Plesiomonas* was significantly reduced with a 45% level of substitution. In addition, TM significantly improved the abundance of bacteria in the Bacilli in the intestinal tract of large yellow croakers, the former of which had been reported to be a probiotic bacteria with beneficial effects on the growth and intestinal health of fish [64,65,66]. Moreover, the 30% and 45% substitution levels significantly reduced the abundance of Peptostreptococcaceae at the family level in the intestine, which was shown to be a potentially pathogenic bacterium that affects metabolic disorders [67]. These data suggest that TM selectively improved the proliferation of the intestinal flora and changed the flora composition of large yellow croakers.

## 5. Conclusions

In summary, the dietary fish meal could be replaced by 15% TM in feeds containing 40% fish meal without adversely affecting the growth of large yellow croakers, and to some extent improving the immunity of the organism. Substitution levels of 15% or more are beneficial for digestive enzymes. In addition, the moderate addition of TM improves intestinal health by improving the structure and microbial composition of the gut.

## Figures and Tables

**Table 1:** Nutrient composition of FM and TM used in this study (% dry matter).

Ingredients (%)	Protein Sources	
	FM	TM
Aspartic acid	6.20	4.61
Glutamic acid	9.45	6.93
Serine	2.02	4.91
Histidine	1.82	0.65
Glycine	4.01	4.67
Threonine	2.33	2.30
Arginine	3.87	3.93
Alanine	4.11	3.09
Tyrosine	1.78	1.88
Cystine	0.52	2.14
Valine	3.54	3.99
Methionine	1.67	1.16
Phenylalanine	3.09	3.01
Leucine	2.85	4.74
Isoleucine	4.86	2.65
Lysine	4.78	4.89
Proline	2.55	5.95
Tryptophan	0.57	0.39
Total	60.02	61.89
Proximate analysis		
Crude protein	68.00	63.84
Crude lipid	9.66	1.99

FM: fish meal; TM: *Tenebrio molitor* meal.

**Table 3:** Growth, feed utilization, morphological parameters and nutrient depositions of large yellow croakers fed diets with different levels of TM.

	Experimental Diets			
	TM0	TM15	TM30	TM45
SR (%)	84.22 ± 3.86	89.33 ± 3.06	76.33 ± 4.43	76.67 ± 1.15
IBW (g)	11.76 ± 0.06	11.78 ± 0.06	11.84 ± 0.02	11.84 ± 0.04
FBW (g)	26.59 ± 0.27 ^b^	26.18 ± 0.31 ^b^	24.89 ± 0.15 ^b^	21.31 ± 1.02 ^a^
WG (%)	124.92 ± 2.30 ^b^	121.47 ± 2.67 ^b^	110.33 ± 1.23 ^b^	80.29 ± 8.66 ^a^
FE	0.72 ± 0.02 ^b^	0.70 ± 0.01 ^b^	0.57 ± 0.04 ^a^	0.47 ± 0.03 ^a^
FI (%/d)	2.12 ± 0.08	2.00 ± 0.06	2.24 ± 0.14	2.36 ± 0.04
PER	1.50 ± 0.04 ^c^	1.44 ± 0.02 ^bc^	1.20 ± 0.08 ^ab^	0.98 ± 0.05 ^a^
CF (g cm^−3^)	1.91 ± 0.03	1.83 ± 0.03	1.83 ± 0.03	1.81 ± 0.03
HSI (%)	2.24 ± 0.17	2.59 ± 0.01	2.61 ± 0.04	2.51 ± 0.06
VSI (%)	7.22 ± 0.16	7.63 ± 0.09	7.62 ± 0.05	7.55 ± 0.09
PDR (%)	4.19 ± 0.08 ^b^	3.92 ± 0.03 ^b^	3.65 ± 0.15 ^b^	2.61 ± 0.26 ^a^
LDR (%)	17.80 ± 0.85 ^c^	15.50 ± 0.48 ^bc^	13.70 ± 0.06 ^b^	10.75 ± 0.56 ^a^

In the same row, values with different superscripts (a, b, c) indicate significant difference (*p* < 0.05). Abbreviations: SR, survival rate; IBW, initial body weight; FBW, final body weight; WG, weight gain rate; FE, feed efficiency; FI, feed intake; PER, protein efficiency ratio; CF, condition factor; HSI, hepato-somatic index; VSI, viscera-somatic index; PDR, protein deposition rate; LDR, lipid deposition rate. TM, *Tenebrio molitor* meal.

**Table 4:** Body compositions of large yellow croakers fed diets with different levels of TM.

Index (Wet Weight, %)	Experimental Diets			
	TM0	TM15	TM30	TM45
Whole-body				
Moisture	74.16 ± 0.18 ^a^	74.68 ± 0.38 ^ab^	75.71 ± 0.41 ^ab^	75.91 ± 0.38 ^b^
Crude protein	13.65 ± 0.22	13.44 ± 0.14	13.59 ± 0.25	13.56 ± 0.06
Crude lipid	7.71 ± 0.26	7.34 ± 0.23	7.17 ± 0.02	7.19 ± 0.10
Ash	3.18 ± 0.08 ^a^	3.16 ± 0.01 ^a^	3.38 ± 0.02 ^b^	3.31 ± 0.02 ^ab^
Liver				
Crude lipid	19.00 ± 0.27 ^b^	17.27 ± 0.22 ^a^	19.20 ± 0.19 ^b^	19.17 ± 0.25 ^b^

In the same row, values with different superscripts (a, b) indicate significant difference (*p* < 0.05). TM, *Tenebrio molitor* meal.

**Table 5:** Serum immunity indexes of large yellow croakers fed diets with different levels of TM.

Index	Experimental Diets			
	TM0	TM15	TM30	TM45
AKP (U/mL)	25.65 ± 0.47 ^bc^	25.36 ± 1.15 ^c^	18.39 ± 0.86 ^b^	14.13 ± 0.74 ^a^
LZM (μg/mL)	4.96 ± 0.18 ^a^	5.67 ± 0.11 ^b^	5.33 ± 0.04 ^ab^	5.29 ± 0.22 ^ab^
C3 (μg/mL)	208.03 ± 7.42 ^a^	211.76 ± 9.82 ^a^	256.64 ± 11.15 ^b^	224.57 ± 8.03 ^ab^
C4 (μg/mL)	1099.97 ± 25.43 ^c^	569.28 ± 19.03 ^b^	508.75 ± 7.34 ^b^	350.77 ± 12.16 ^a^

In the same row, values with different superscripts (a, b, c) indicate a significant difference (*p* < 0.05). Abbreviations: AKP, alkaline phosphatase; LZM, lysozyme; C3, complement 3; C4, complement 4. TM, *Tenebrio molitor* meal.

## Data Availability

The data presented in this study are available on request from the corresponding author.
